# Mercy Hospital

**Published:** 1878-09

**Authors:** F. E. Waxham


### Clinical Reports.

### MERCY HOSPITAL.

*Two Cases of Lithotomy, Performed by Dr. Andrews, June 13th.*

REPORTED BY F. E. WAXHAM, M. D.

Case 1. E. R. H., æt 30. Entered the hospital June 5th, 1878. The patient was very much emaciated, and his lungs affected with tuberculosis. He stated that he had cystitis of six years' standing, which commenced with pain in the bladder, and a frequent desire to urinate. The disease increased in severity for a year, when he began to submit himself to treatment; he then gradually improved. The sound was introduced into the bladder at two different times, but no calculus discovered. He remained quite comfortable until the fall of 1877, when he began to get worse, and was obliged to give up work. The pain left the bladder and had centered in the urethra, which became extremely sensitive. Upon entering the hospital, he was obliged to pass water every few moments, in great pain, the urine containing a large amount of mucus and pus. The urethra was so sensitive that the softest flexible catheter could not be introduced into the bladder. Soothing injections were frequently practiced in the urethra to control the hyperæsthesia. As he could not endure the introduction of a sound, he was anæsthetized with ether, June 13th. A partial stricture was discovered about five inches from the meatus; this was dilated, and after much difficulty a sound introduced into the bladder. A calculus being found, lithotomy was at once decided upon, as it was not considered a favorable case for lithotrity on account of the great sensitiveness of the urethra. The patient was placed in the lithotomy position, but it was found impossible to introduce the staff. A long beaked instrument could be passed into the bladder, but one with a short curve could not. The sound was introduced and the incision made upon it using a grooved director in conjunction with it in place of the staff. The calculus was removed without difficulty, and weighed 8 grams. After recovering from the anæsthetic he suffered much pain, and required large doses of opium to obtain relief. The intense pain and frequent desire to pass water, soon subsided, and the patient gradually improved, although the wound has been slow in healing, a portion of the urine still passing through the wound. The patient left the hospital July 22. Time in hospital, 47 days.

Case 2. Edward Cloonan, æt 15 years. Entered the hospital June 12th. Has suffered from pain in the penis, and frequent desire to pass water, for three years. A year ago a sound was introduced into the bladder, and a large stone detected. The father objected to an operation at that time, as he had lost one child only a few months previous, from the same cause, lithotomy having been performed. Upon entering the hospital the patient was in a good condition physically. June 13th, gave ether and performed lithotomy. Great difficulty was experienced in removing the calculus on account of its large size. The tissues were bruised and lacerated to a considerable extent. The calculus measured 7 centimeters in length and 4½ centimeters in thickness, and weighed 72 grams. Notwithstanding the unfavorable prognosis, on account of the contusion of the tissues, he made a rapid recovery, suffering no pain and requiring no anodynes. Patient left the hospital July 13th. The wound was entirely closed, and the urine passed freely through the penis. Time in hospital, 31 days.

